# Protective Effect of Tomato By-Product in Refined Sunflower Oil with Different Lipid Profiles

**DOI:** 10.3390/molecules30142968

**Published:** 2025-07-15

**Authors:** Idoya Fernández-Pan, Sandra Horvitz, Francisco C. Ibañez, Paloma Vírseda, María José Beriain

**Affiliations:** Institute for Sustainability & Food Chain Innovation—ISFOOD, Universidad Pública de Navarra, Campus Arrosadia s/n, 31006 Pamplona, Spain; idoya.fernandez@unavarra.es (I.F.-P.); sandra.horvitz@unavarra.es (S.H.); pi@unavarra.es (F.C.I.); mjberiain@unavarra.es (M.J.B.)

**Keywords:** tomato waste, vegetable oil, high-pressure processing, oleic acid content, lycopene, carotenoids, tocopherols, thermostability

## Abstract

The recovery of carotenoids, particularly lycopene, from industrial tomato by-products is contingent upon the composition of the raw material, the harvesting season, and the specifics of the extraction process. Industrial tomato by-product from three harvest seasons (S1, S2, and S3) was revalorized and used as a lycopene natural source. Pressurization-assisted extraction of lycopene was carried out using two types of refined sunflower oil (high oleic, HO, and low oleic, LO). The carotenoid and tocopherol content, as well as the fatty acid profile, were analyzed in the resulting HO and LO oil samples, and thermooxidation stability was evaluated. Lycopene recovery was found to be higher in the LO oil than in the HO oil using the by-product from the S3 harvest. Conversely, the tocopherol content declined in both oil types following the incorporation of the S3 by-products. The addition of by-products did not affect the thermooxidation stability of the HO oil. Conversely, the thermooxidation stability of the LO oil increased by about 3.2 ± 0.6 h, irrespective of the season. The findings of this study demonstrate that the addition of tomato by-product, regardless of its lycopene content, provides a protective effect against the thermooxidation of conventional sunflower oil.

## 1. Introduction

The global production of fresh tomatoes (*Lycopersicon esculentum*) reaches annually around 180 million tons, with approximately 40.5 tons grown for the processing industry in 2024 [1]. The industrial processing of tomatoes generates high quantities of by-products, known as tomato pomace, which can represent up to 10% of the whole fresh produce [2]. These residues contain different amounts of peel, pulp and seeds and are rich in proteins, dietary fiber, minerals, and biologically active compounds, including carotenoids, with lycopene being the most important one followed by β-carotene [3,4]. The content of lycopene in tomato by-products is highly variable, and is influenced by genetics and meteorological conditions during the growing season [5]. In particular, temperatures above 30 °C and dryness can inhibit lycopene biosynthesis [6], although these effects are cultivar dependent [7]. Precisely because of these variations, it is of the outmost relevance for industrial valorization and application purposes to select by-products that allow for obtaining extraction yields that technically and economically justify the processing.

Lycopene recovery from tomato pomace is typically performed by using mixtures of organic solvents in conjunction with physical technologies to disrupt the cell membranes [3]. However, this method requires long extraction times and high energy inputs, and generates extracts with organic solvent residues that are usually toxic and, thus, need to be eliminated [8].

Consumers’ concerns about sustainability and health, and the increasing interest in recovering lycopene from natural sources have driven research into the use of green technologies [9,10]. Considering the lipophilic nature of lycopene, edible vegetable oil has been studied for the extraction of lycopene. These types of oil are regarded as eco-friendly solvents as they are safe and do not need to be eliminated following extraction. Additionally, lycopene extracts can protect the oil from oxidation [11]. However, the influence of the oil’s fatty acid profile on lycopene recovery and its potential protective effect against thermooxidation has not yet been thoroughly evaluated. Together with these green solvents, technologies such as ultrasound, microwaves, pulsed electric fields, and high-pressure processing (HPP), among others, have been evaluated to weaken the cell membranes and consequently, enhance the recovery of lycopene from tomato by-products. HPP has an advantage in terms of reducing the extraction time of lycopene and improving the efficiency of the recovery process [12].

In this context, refined sunflower oil (SO) might constitute an interesting proposal to be used as a green solvent. It is one of the most important types of edible oil for human nutrition and it is widely available, ranking fourth among the most produced vegetable oil worldwide [9,13]. Conventional SO is moderate in monounsaturated and rich in polyunsaturated fatty acids, including oleic (14–40%) and linoleic (40–74%) acids, respectively. This fatty acid profile renders SO more susceptible to oxidation and/or degradation [14]. On the other hand, in high oleic SO, the content in oleic acid is up to four times greater and can reach 84% acid in oil [15,16]. Due to its good technological properties and low saturated fats content, this oil is demanded by the food industries to replace *trans*-fats [17].

The objectives of this study are as follows: (i) to identify and quantify the biomolecules recovered from industrial tomato by-products of different harvesting seasons using refined conventional low oleic (LO) and high oleic (HO) sunflower oil as green solvents; and (ii) to analyze the thermooxidation stability of the lycopene-enriched LO and HO oil.

## 2. Results

### 2.1. Tomato By-Product Composition and Recovery of Bioactive Molecules

The proximal composition of the industrial tomato by-product, made up of peels and seeds, is presented in Table A1.

The recovery of lycopene and carotenoids from tomato by-products using the LO and HO sunflower oil samples was assisted by a high-pressure processing settled at 450 MPa for 10 min. Internal probes indicated that processing was performed within a range of 447–453 MPa, and a thermal jump of 10–15 °C ensuring that the processed samples remained below 40 °C. It is important to remark that temperature plays a critical role in the extraction and stabilization of carotenoids, with lycopene being a notable example.

### 2.2. Physical–Chemical Analyses

Acidity, expressed as a percentage of oleic acid, ranged from 0.05 (HOu-C and LOu-C) to 0.07% (oil enriched with tomato by-product). No statistically significant differences were detected among the samples.

The carotenoid content of the unenriched and enriched with tomato by-product SO samples is presented in Table 1. In all the enriched samples, *trans*-lycopene was the predominant carotenoid, and the observed concentrations are in accordance with the lycopene content of the tomato by-product extracts (Table A1). Statistically significant differences were detected as a function of the harvesting season and the type of SO, with the highest levels found in the LO oil samples from seasons 2 and 3.

The instrumental color parameters of the SO containing tomato by-product are summarized in Table 2. The brightest samples were those of the LO oil control and those enriched with tomato by-product from season 1. The darkest samples were found in the LO oil from seasons 2 and 3 and the HO from season 3. The most reddish and yellowish samples were those of the LO and HO oil samples with tomato by-product from season 3. These outcomes are in alignment with the carotenoid content, notably lycopene, present in the samples (Table 1).

**Figure 1:**
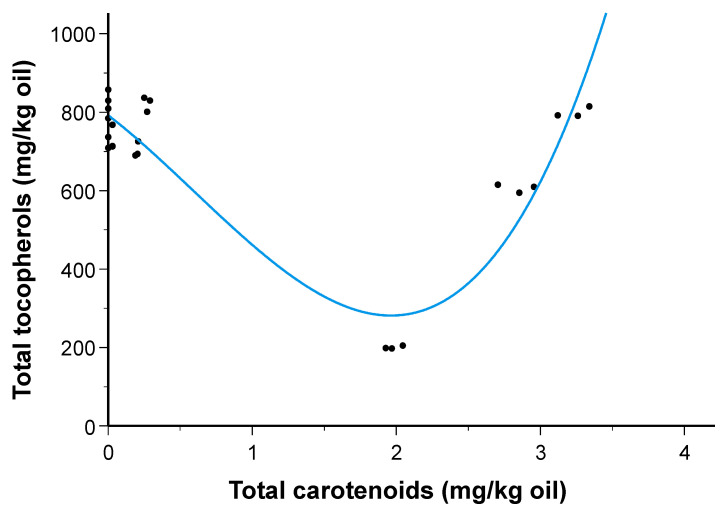
Total tocopherol content in sunflower oil as a function of total carotenoid content from tomato by-product (irrespective of oil type and tomato harvesting season). Dots represent the experimental values.

The individual and total tocopherol contents of the oil samples are presented in Table 3. A progressive decrease in the total content of these molecules was detected in both types of oil, from season 1 to season 3. This reduction was especially noticeable in the HO oil samples with the α-tocopherol content of the HOp-S3 sample decreasing by 77.9% and 77.3% in comparison to the unpressurized and pressurized control samples, respectively. On the assumption that a relationship exists between total tocopherol content and total carotenoid content, regardless of tomato harvesting season and oil type, a nonlinear regression model can be proposed to predict the tocopherol content as follows:

*E* = 762.3 − 282.1 × *C* − 110.1 × *C*^2^ + 61.8 × *C*^3^; *R* = 0.918 (1)

where *E* is the total tocopherol content (mg/kg oil) and *C* is the total carotenoid content (mg/kg oil). The regression model explained 83% of the variability in the tocopherol content as a function of the carotenoid content (Figure 1). The minimum tocopherol content was reached when the carotenoid contents were close to 2 mg/kg oil.

Table 4 provides a summary of the fatty acid profile, expressed as a percentage of total oil weight. The differences in the saturated and monounsaturated fatty acid profiles are in accordance with the type of SO, whether it is conventional or high oleic. Globally, the LO oil samples presented higher contents of saturated fatty acids compared to the HO oil samples, with no significant differences within each group. The predominant acids in both types of oil samples were palmitic (C16:0) and stearic (C18:0) acids, with significantly higher contents of these fatty acids in the LO oil samples. On the contrary, the content of arachidic (C22:0) acid was found to be higher in the HO oil samples in comparison with the LO oil samples. Pressurization did not impact on the saturated fatty acid (SAFA) contents of both oil types.

The oleic (C18:1) and 9-eicosenoic (C20:1) acids contents were, respectively, 56.22 and 0.10 percentage points higher in the HO oil samples compared with the LO oil samples. No statistically significant differences in the monounsaturated fatty acid (MUFA) content were found between the pressurized and non-pressurized samples. Regarding the polyunsaturated fatty acids, the LO oil samples presented 10 times more *cis*-linoleic acid (C18:2) than the HO oil samples, with no significant differences due to pressurization. On the contrary, the contents of *trans*-C18:2 and *cis*-C18:3 acids in the low oleic oil samples were significantly lower in the pressurized samples. However, this effect was not observed in the high oleic oil samples.

### 2.3. Thermooxidative Stability

**Figure 2:**
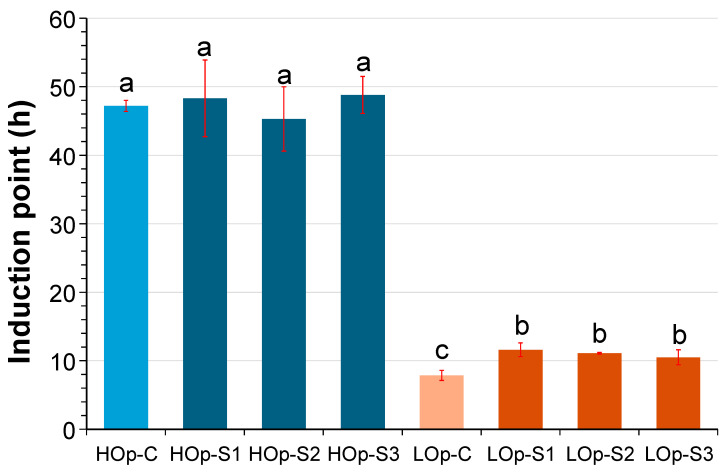
Thermal stability of refined sunflower oil (HOp: pressurized high oleic; LOp: pressurized low oleic) unenriched (C) or enriched with tomato waste from three harvesting seasons (S1, S2, and S3). Data are expressed as mean ± standard deviation (*n* = 3). Different letters indicate significant differences (*p* < 0.05) per Tukey’s test.

Figure 2 illustrates the changes in the thermooxidative stability of the SO samples when subjected to heating at 90 °C in a high oxidative stress atmosphere. No significant differences due to the growing season were observed in either oil. On the other hand, the HO oil samples presented higher induction points when compared with the LO oil samples. Furthermore, in the former, no statistically significant differences were detected among the samples due to tomato by-product addition. Conversely, in the low oleic oil samples (LO), those containing tomato by-product exhibited higher induction point values compared to the samples devoid of added by-product (LOp-C).

## 3. Discussion

The efficiency of lycopene extraction from tomato peels and seeds using edible vegetable oil with different lipid profiles depends on the appropriate combination of oil samples and process parameters for specific operating conditions. Regarding the type of oil used as green solvent, Kunthakudee et al. [18] reported that sunflower oil (low oleic) was more effective than olive oil (high oleic) in recovering lycopene from tomato skin residues. On the contrary, Nour et al. [11] and Benakmoum et al. [19] found similar results when comparing sunflower and extra virgin olive oil as green solvents for extracting lycopene from tomato skins. The variability in lycopene and other carotenoid contents in tomato by-products, depending on the harvesting season, has been reported to be considerable and can be explained by differences in both agricultural practices and weather conditions during the growing season [20,21].

Considering high-pressure, different process conditions have been evaluated to facilitate lycopene recovery from tomato paste waste. By adjusting the P-t variables (100–600 MPa; 1–10 min), the solvent type (e.g. chloroform, ethanol, edible oil, or water) and the solid/liquid ratio (1:1 to 1:8 g/mL), it was possible to reduce the conventional processing times. Applying 500 MPa for 1 min with 75% ethanol as the solvent in 1:5 [22] and 1:6 [23] ratios resulted in the recovery of over 90% of the lycopene from tomato skins and seeds. With a similar type of tomato by-product, Fernández-Pan et al. [24] tested refined sunflower oil and extra-virgin olive oil as green solvents for lycopene and carotenoids recovery assisted by high pressures of 300 to 600 MPa for 10 min. These authors found that, for a fixed by-product/oil ratio, olive oil was the most suitable solvent because of the higher recovery yield.

To date, no studies exploring the impact of vegetable oil pressurization on tocopherol content have been published. Decreases in tocopherol content have been reported in beverages derived from juice or milk when subjected to high-pressure processing, ranging from 100 to 400 MPa [25]. Also, the impact of high-pressure processing (100–600 MPa) has been investigated in the recovery of biomolecules embedded in oil/water emulsions. A decline in the contents of β-carotene and α-tocopherol was detected following treatment at 400 MPa [26]. In the present study, it was detected that the amount of tocopherols was lower for a given concentration of carotenoids. The findings suggest a non-linear relationship between the potential degradation rate of tocopherol molecules and the presence of carotenoid molecules within a pressurized oil medium. Nevertheless, the oil samples enriched with tomato by-products were found to contain very low levels of polyphenols. The values obtained in all the samples were lower than 0.005 mg of caffeic acid equivalent/g of oil. These results were consistent with those reported by Benakmoum et al. [19]. These researchers pointed out that the incorporation of tomato puree in amounts higher than 20% was required to increase the phenolic content in standard sunflower oil. The low solubility of polyphenols in lipids, attributable to their abundant phenolic hydroxyl groups, leads to a certain restriction in their incorporation into edible oil [27].

The high concentration of polyunsaturated fatty acids (PUFA) present in the SO makes these types of oil prone to oxidation. Thus, several studies have focused on the supplementation with natural antioxidants to stabilize refined sunflower oil [28]. The effects of lycopene enrichment on the thermostability of SO varied with the type of oil used. In the LO-refined sunflower oil, the incorporation of lycopene and carotenoids significantly improved the oxidative stability. These results agree with Gheonea et al. [29], who identified an improvement in the oxidative stability of lycopene-rich peanut and cotton oil, with a 30% oleic acid content, which is similar to the oleic acid concentration found in the LO-SO in the present study. However, while lycopene provides a protective effect at moderate temperatures in most types of oil, appropriate concentrations and complementary antioxidants are required in some cases to prevent or avoid pro-oxidant effects.

Lycopene and β-carotene react with oxygen to form peroxyl radicals, which may enhance the propagation stage of the oxidation reaction by supplying the system with more oxidizable substrates. In previous studies, it has been reported that an increase in carotenoid concentration induced a pro-oxidant effect of lycopene and β-carotene in soybean triacylglycerol [30] and of lycopene in safflower oil (usually >70% PUFAs) [31].

According to Zeb and Murkovic [32], increasing β-carotene concentration in corn, rapeseed, and sunflower oil induced more peroxide formation showing that β-carotene or its oxidized species acted as pro-oxidants. Carotenoids can interact with these radicals and subsequently undergo cleavage [33].

In the present study, the proportion of unsaturated fatty acids was similar in both types of oil. Thus, the lowest stability of the LO-SO could be the result of a higher PUFA/MUFA ratio in this oil (1.97 ± 0.03 vs. 0.07 ± 0.00 in the HO-SO) and a higher reactivity of PUFA than of MUFA (α-linolenic acid > linoleic acid > oleic acid), as has previously been shown [34].

On the other hand, the presence of tocopherols may prevent β-carotene and lycopene degradation during lipid peroxidation. Therefore, the higher stability to oxidation in the LO-SO enriched with lycopene, could be due to the protective effect of tocopherols against the auto-oxidation of lycopene by free radicals. In this scenario, the pro-oxidant effect of carotenoids would be inhibited by tocopherols. It is worth considering whether some degree of carotene oxidation might occur during the extraction procedure itself, potentially leading to a subsequent loss of tocopherols. However, it has been reported that, when α-tocopherol amounts significantly exceed those of β-carotene, their interaction yields synergistic effects. This synergy may arise from β-carotene’s primary reaction with free radicals, protecting α-tocopherol from oxidation. Furthermore, the lower reduction potential of α-tocopherol combined with the higher potential of β-carotene facilitates mutual repair and regeneration. Following the oxidation of α-tocopherol to α-tocopherol-quinone, β-carotene can restore it to its active form. Conversely, when the amounts of α-tocopherol and β-carotene are comparable, or when β-carotene predominates, the synergistic effects diminish, leading to potential antagonism. This antagonism may result from hydrogen bonding between the hydroxyl groups of β-carotene and α-tocopherol, impeding the latter’s hydrogen donation ability, or from the oxidation of β-carotene, which reduces the antioxidant efficacy and induces antagonistic interactions [35].

The presence and type of tocopherols directly influence the thermostability of lycopene in vegetable oil, with mostly positive effects that vary depending on the specific oil matrix. Adding tocopherols improves lycopene retention and slows the degradation of SO [36]. Hackett et al. [37] reported that α-tocopherol doubled lycopene’s half-life from 6.1 to 12.5 days at 50 °C in oleoresins prepared with corn oil. Kaur et al. [30] found that a 1:2 ratio of lycopene to γ-tocopherol enhanced the antioxidant efficiency in soybean oil. Furthermore, Zuorro [10] noted that lycopene from tomato peels was more stable in oil matrices naturally rich in tocopherol, such as sunflower seed oil, than in matrices with lower tocopherol levels, such as grape seed oil.

Thus, the related literature suggests that the relationship between lycopene and tocopherols is highly matrix-dependent, and their interaction can be mostly synergistic [36,37,38], but also antagonistic, as lycopene can act as a pro-oxidant depending on the specific conditions and ratios present in the system [30]. In this sense, Kaur et al. [30] also reported that the combination of lycopene and γ-tocopherol (1:2) acted as an antioxidant with better efficiency than γ-tocopherol alone.

Varas-Condori et al. [38] informed that adding tomato lycopene extracts at 80 mg lycopene/kg oil increased linseed oil thermooxidation stability similarly to the addition of 200 mg/kg BHT. In this context, it can be proposed that tomato by-products be used as a natural source of lycopene for improving SO thermostability.

## 4. Materials and Methods

### 4.1. Tomato By-Product Batches, Sample Conditioning and Preparation

Three batches of industrial tomato by-products supplied by the company AN Conservas Dantza© (Castejon, Spain), corresponding to the summer campaigns of 2022 (season 1), 2023 (season 2), and 2024 (season 3) were used in this study. The tomatoes were harvested and processed in September of the same years mentioned above. The tomato by-product was a mixture of skins and seeds obtained during the production of tomato sauce, specifically after the blanching stage. Each year, eighty kg of tomato by-product was received fresh, portioned in 2-kg batches, frozen on the same day of production and kept at –20 °C until use in this study. After 24 h of defrosting at 10 °C, 4 kg of each batch were dried in a convection oven (model D4AFY, Kowell Corp., Gyeongg-i-do, Republic of Korea) at 60 °C for 6 h.

The proximate composition of the tomato by-product from seasons 1 and 2 was presented in previous studies as indicated in Table A1. Table A1 also shows the proximate composition of the tomato by-product obtained in season 3, employing the same analytical methodologies.

### 4.2. Recovery of Bioactive Molecules: Vegetable Oil and Pressurization Treatment

In all cases, the samples were processed using a solid–liquid extraction process assisted by an IDUS25L003 high pressure equipment (Idus HPP Systems S.L.U., Noain, Spain) using two types of refined SO as green solvents.

The selected refined SO (Urzante SL©, Tudela, Spain) was BHT-free and differed in its oleic acid content: high oleic (HO) and low oleic (LO). For the extraction process, samples of each oil were prepared in polypropylene bags by adding 50 g of dried tomato by-product from each season to 500 mL of oil (10%, *w/v*). Control samples consisted of oil without the addition of tomato by-product. The bags were heat sealed and subjected to high-pressure processing.

In all cases, the same pressurizing treatment was applied, fixed at 450 MPa for 10 min and room temperature (20 ± 5 °C). To monitor the extraction processes, the equipment’s own software was used to record pressure and time data. Additionally, an extra bag containing a temperature probe protected by a steel case specifically designed for the probe to withstand pressure, was introduced for each test and the internal temperature of the samples was recorded.

After processing, the tomato by-product was immediately separated and discarded, and the resulting oil samples were kept in polypropylene tubes at room temperature (20 ± 2 °C) and in the dark until analysis.

### 4.3. Physical–Chemical Analyses of Vegetable Oil

After processing, the following parameters were determined in the oil samples: color, acidity, fatty acids composition, and carotenoid, tocopherol, and total polyphenol contents. The standard methods used for these determinations are referenced in Table A2. The fatty acid composition was determined using a gas chromatographic device (7890B Agilent Technologies, Inc., Santa Clara, CA, USA) with an FID detector. The experimental procedure of Salas et al. [39] was followed. The analysis of tocopherols was conducted using a 1260 Infinity II HPLC system with a fluorescent detector (Agilent Technologies, Inc., Santa Clara, CA, USA), in accordance with the conditions set by Velasco et al. [40]. An HPLC device (HP 1100, Hewlett-Packard Inc., Palo Alto, CA, USA) fitted with a diode array detector was used for the carotenoid determination, following the method described by Gandul-Rojas and Gallardo-Guerrero [41]. The total polyphenol content was estimated using the method proposed by Vazquez-Roncero et al. [42], and the results were expressed as milligrams of caffeic acid equivalents per gram of oil.

### 4.4. Thermal Oxidative Stability

The stability to thermooxidation of the samples was determined in an OXITEST^®^ reactor (Velp Scientifica SrL, Usmate, Italy). Following the manufacturer’s indications, 5 g of each oil was fitted in each of the oxidation chambers and subjected to an oxidative stress environment under conditions of high temperature (90 °C) and high oxygen pressure (6 bar). The pressure inside the chambers was registered every minute, and the analyses continued until a drop in oxygen pressure inside the chambers was detected. The time required for this rapid change in the oxidation rate to occur is expressed as the induction period, and was obtained using the two-tangent method. Each oil sample was analyzed in triplicate, and the results are expressed in hours.

### 4.5. Data Processing and Statistical Analyses

Data processing and statistical analyses were conducted according to the requirements of the official methods indicated in Table A2 and using the SPSS statistical software Statistics for Windows, vers. 28.0 (IBM Corp., Armonk, NY, USA). The oil type × season effects were evaluated using a two-way ANOVA. When the interaction term was not significant, a one-way ANOVA was employed. Differences between the pairwise of means were tested using Tukey’s test (95% significance level). A regression analysis was conducted to explore the predictive model for tocopherol content as a function of carotenoid content.

## 5. Conclusions

Refined sunflower oil with different lipid profiles acted as an effective green solvent in the HPP-assisted treatment of industrial tomato by-products, recovering valuable biocompounds, such as carotenoids and, specifically, lycopene. *Trans*-lycopene was the predominant carotenoid in all the cases, and the carotenoid content of the resulting sunflower oil depended on the tomato growing season. Considering the oil type, the concentration of the carotenoids was higher in the low oleic oil than in the high oleic oil. The thermooxidation stability of high oleic sunflower oil was unaffected by the addition of tomato by-product as a source of lycopene. However, when added to low oleic sunflower oil, it enhanced its thermooxidation stability, regardless of the amount of lycopene incorporated. This study shows that lycopene added to conventional sunflower oil in a revalorization process of industrial tomato by-product can protect the oil, having relevant implications considering its shelf life and quality composition. Therefore, it can be stated that industrial tomato by-products, rich in carotenoids, can act as a natural source of lycopene to be used as an antioxidant for conventional sunflower oil to improve their thermostability.

## Figures and Tables

**Table 1:** Contents of carotenoids (mg/kg oil) in refined sunflower oil (HOp: pressurized high oleic; LOp: pressurized low oleic) and unenriched (C) or enriched with tomato by-product from three harvesting seasons (S1, S2, and S3). Data are expressed as mean ± SD (*n* = 3).

Carotenoid	HOp-C	HOp-S1	HOp-S2	HOp-S3	LOp-C	LOp-S1	LOp-S2	LOp-S3
*cis*-lycopene	0.00 ± 0.00 ^c^	0.00 ± 0.00 ^c^	0.01 ± 0.01 ^c^	0.09 ± 0.00 ^b^	0.00 ± 0.00 ^c^	0.00 ± 0.00 ^c^	0.13 ± 0.03 ^a^	0.14 ± 0.02 ^a^
*trans*-lycopene	0.00 ± 0.00 ^e^	0.03 ± 0.01 ^e^	0.18 ± 0.02 ^de^	1.80 ± 0.07 ^c^	0.00 ± 0.00 ^e^	0.27 ± 0.03 ^d^	2.96 ± 0.16 ^a^	2.56 ± 0.14 ^b^
*trans*-β-carotene	0.00 ± 0.00 ^d^	0.00 ± 0.00 ^d^	0.01 ± 0.01 ^d^	0.09 ± 0.00 ^c^	0.00 ± 0.00 ^d^	0.01 ± 0.01 ^d^	0.15 ± 0.02 ^a^	0.13 ± 0.01 ^b^
*trans*-lutein	0.00 ± 0.00 ^b^	0.00 ± 0.00 ^b^	0.00 ± 0.00 ^b^	0.00 ± 0.00 ^b^	0.00 ± 0.00 ^b^	0.00 ± 0.00 ^b^	0.01 ± 0.01 ^a^	0.01 ± 0.01 ^a^

Different letters in the same row indicate significant differences (*p* < 0.05) per Tukey’s test.

**Table 2:** Coordinates of instrumental color for refined sunflower oil (HOp: pressurized high oleic; LOp: pressurized low oleic), and unenriched (C) or enriched with tomato by-product from three harvesting seasons (S1, S2, and S3). Data are expressed as mean ± SD (*n* = 3).

Parameter	HOp-C	HOp-S1	HOp-S2	HOp-S3	LOp-C	LOp-S1	LOp-S2	LOp-S3
L*	39.35 ± 1.45 ^bc^	39.75 ± 1.31 ^bc^	37.66 ± 0.19 ^c^	31.92 ± 1.19 ^d^	43.32 ± 0.07 ^a^	41.31 ± 0.51 ^ab^	34.39 ± 0.37 ^d^	34.24 ± 1.49 ^d^
a*	−1.83 ± 0.07 ^d^	5.18 ± 0.06 ^c^	15.03 ± 0.11 ^b^	26.65 ± 3.14 ^a^	−1.73 ± 0.04 ^d^	3.78 ± 0.11 ^c^	16.1 ± 0.19 ^b^	28.51 ± 1.51 ^a^
b*	10.73 ± 0.13 ^d^	18.98 ± 0.60 ^c^	22.82 ± 0.17 ^b^	38.47 ± 2.41 ^a^	12.31 ± 0.10 ^d^	19.64 ± 0.22 ^c^	24.1 ± 0.19 ^b^	37.6 ± 1.85 ^a^

Different letters in the same row indicate significant differences (*p* < 0.05) per Tukey’s test.

**Table 3:** Contents of tocopherols (mg/kg oil) in refined sunflower oil (HOp: pressurized high oleic; LOp: pressurized low oleic) and unenriched (C) or enriched by tomato waste from three harvesting seasons (S1, S2, and S3). Data are expressed as mean ± SD (*n* = 3).

Tocopherol	HOu-C	HOp-C	HOp-S1	HOp-S2	HOp-S3	LOu-C	LOp-C	LOp-S1	LOp-S2	LOp-S3
α	736.4 ± 23.6 ^abc^	712.7 ± 34.9 ^bcd^	701.4 ± 29.2 ^bcd^	676.7 ± 16.8 ^d^	162.0 ± 3.7 ^f^	804.0 ± 24.7 ^a^	747.0 ± 24.9 ^ab^	778.3 ± 22.5 ^ab^	759.3 ± 11.0 ^abc^	578.7 ± 12.0 ^e^
β	27.4 ± 1.6 ^abc^	27.0 ± 2.7 ^abc^	26.7 ± 2.1 ^abc^	24.4 ± 4.1 ^c^	29.7 ± 0.6 ^abc^	27.4 ± 1.6 ^abc^	26.8 ± 1.5 ^abc^	30.3 ± 2.4 ^ab^	32.3 ± 1.8 ^a^	24.7 ± 2.1 ^bc^
γ	4.0 ± 0.1 ^cd^	4.0 ± 1.0 ^cd^	3.7 ± 0.6 ^cd^	2.0 ± 1.0 ^d^	9.0 ± 0.1 ^a^	1.3 ± 0.2 ^e^	5.0 ± 2.2 ^bc^	8.0 ± 2.5 ^ab^	8.3 ± 0.5 ^ab^	3.4 ± 0.6 ^cd^
total	767.7 ± 25.1 ^abc^	743.7 ± 38.5 ^abc^	731.7 ± 31.5 ^bc^	700.4 ± 21.3 ^c^	200.4 ± 4.2 ^e^	831.4 ± 26.8 ^a^	778.8 ± 29.1 ^ab^	816.5 ± 19.9 ^a^	799.8 ± 12.7 ^ab^	606.7 ± 10.5 ^d^

Different letters in the same row indicate significant differences (*p* < 0.05) per Tukey’s test.

**Table 4:** Fatty acid profile for refined sunflower oil (HO: high oleic; LO: low oleic) and unenriched (C) or enriched with tomato waste from three harvesting seasons (S1, S2, and S3). Unpressurized samples are coded with “u” and pressurized samples with “p”. Data are expressed as a percentage of total oil weight (mean ± SD; *n* = 3).

(a) Saturated fatty acids										
Acid	HOu-C	HOp-C	HOp-S1	HOp-S2	HOp-S3	LOu-C	LOp-C	LOp-S1	LOp-S2	LOp-S3
C14:0	0.04 ± 0.01 ^b^	0.04 ± 0.01 ^b^	0.04 ± 0.01 ^b^	0.04 ± 0.01 ^b^	0.04 ± 0.01 ^b^	0.08 ± 0.01 ^a^	0.08 ± 0.01 ^a^	0.08 ± 0.01 ^a^	0.08 ± 0.01 ^a^	0.07 ± 0.01 ^a^
C16:0	3.59 ± 0.02 ^b^	3.59 ± 0.23 ^b^	3.60 ± 0.22 ^b^	3.59 ± 0.22 ^b^	3.60 ± 0.02 ^b^	6.32 ± 0.32 ^a^	6.24 ± 0.02 ^a^	6.32 ± 0.32 ^a^	6.32 ± 0.32 ^a^	6.25 ± 0.02 ^a^
C17:0	0.03 ± 0.01	0.03 ± 0.01	0.03 ± 0.01	0.03 ± 0.01	0.03 ± 0.01	0.04 ± 0.02	0.04 ± 0.01	0.03 ± 0.01	0.04 ± 0.02	0.03 ± 0.01
C18:0	2.80 ± 0.13 ^b^	2.80 ± 0.02 ^b^	2.81 ± 0.01 ^b^	2.80 ± 0.14 ^b^	2.80 ± 0.13 ^b^	3.34 ± 0.13 ^a^	3.40 ± 0.01 ^a^	3.33 ± 0.13 ^a^	3.33 ± 0.13 ^a^	3.40 ± 0.01 ^a^
C20:0	0.25 ± 0.03	0.25 ± 0.03	0.25 ± 0.01	0.26 ± 0.01	0.26 ± 0.01	0.25 ± 0.03	0.24 ± 0.04	0.24 ± 0.03	0.24 ± 0.03	0.24 ± 0.03
C22:0	0.86 ± 0.01 ^a^	0.87 ± 0.01 ^a^	0.87 ± 0.01 ^a^	0.88 ± 0.01 ^a^	0.87 ± 0.01 ^a^	0.69 ± 0.05 ^b^	0.71 ± 0.02 ^b^	0.72 ± 0.04 ^b^	0.72 ± 0.04 ^b^	0.71 ± 0.01 ^b^
C24:0	0.31 ± 0.01	0.31 ± 0.01	0.31 ± 0.08	0.31 ± 0.07	0.31 ± 0.08	0.25 ± 0.05	0.25 ± 0.01	0.26 ± 0.05	0.25 ± 0.05	0.25 ± 0.01
Ʃ SAFA	7.88 ± 0.14 ^b^	7.89 ± 0.26 ^b^	7.90 ± 0.31 ^b^	7.91 ± 0.21 ^b^	7.90 ± 0.11 ^b^	10.93 ± 0.31 ^a^	10.94 ± 0.05 ^a^	10.96 ± 0.32 ^a^	10.97 ± 0.15 ^a^	10.94 ± 0.07 ^a^
(b) Monounsaturated fatty acids										
C16:1	0.14 ± 0.01	0.14 ± 0.01	0.15 ± 0.01	0.14 ± 0.01	0.15 ± 0.01	0.13 ± 0.02	0.12 ± 0.01	0.13 ± 0.01	0.13 ± 0.02	0.12 ± 0.01
C17:1	0.15 ± 0.13	0.04 ± 0.01	0.04 ± 0.01	0.04 ± 0.01	0.04 ± 0.01	0.04 ± 0.01	0.03 ± 0.01	0.03 ± 0.01	0.03 ± 0.01	0.04 ± 0.01
*cis*-C18:1	85.95 ± 0.01 ^a^	85.95 ± 0.01 ^a^	85.91 ± 0.01 ^a^	85.95 ± 0.02 ^a^	86.01 ± 0.08 ^a^	29.55 ± 0.83 ^b^	29.59 ± 0.05 ^b^	29.57 ± 0.82 ^b^	29.57 ± 0.84 ^b^	30.32 ± 0.13 ^b^
*trans*-C18:1	0.02 ± 0.01	0.02 ± 0.01	0.02 ± 0.01	0.02 ± 0.01	0.02 ± 0.01	0.03 ± 0.02	0.02 ± 0.01	0.03 ± 0.01	0.04 ± 0.02	0.02 ± 0.01
C20:1	0.25 ± 0.02 ^a^	0.26 ± 0.02 ^a^	0.26 ± 0.02 ^a^	0.26 ± 0.03 ^a^	0.25 ± 0.03 ^a^	0.16 ± 0.01 ^b^	0.15 ± 0.01 ^b^	0.16 ± 0.01 ^b^	0.15 ± 0.01 ^b^	0.15 ± 0.01 ^b^
Ʃ MUFA	86.48 ± 0.13 ^a^	86.40 ± 0.02 ^a^	86.36 ± 0.02 ^a^	86.39 ± 0.04 ^a^	86.46 ± 0.08 ^a^	29.89 ± 0.83 ^b^	29.89 ± 0.05 ^b^	29.90 ± 0.83 ^b^	29.90 ± 0.85 ^b^	30.64 ± 0.11 ^b^
(c) Polyunsaturated fatty acids										
*cis*-C18:2	5.68 ± 0.01 ^b^	5.67 ± 0.01 ^b^	5.69 ± 0.02 ^b^	5.65 ± 0.01 ^b^	5.59 ± 0.07 ^b^	59.12 ± 1.57 ^a^	59.13 ± 0.05 ^a^	59.08 ± 1.55 ^a^	59.08 ± 1.57 ^a^	58.37 ± 0.17 ^a^
*trans*-C18:2	0.08 ± 0.01 ^b^	0.08 ± 0.01 ^b^	0.08 ± 0.01 ^b^	0.08 ± 0.01 ^b^	0.08 ± 0.01 ^b^	0.13 ± 0.04 ^a^	0.08 ± 0.01 ^b^	0.12 ± 0.03 ^ab^	0.12 ± 0.03 ^ab^	0.09 ± 0.01 ^b^
*cis*-C18:3	0.08 ± 0.01 ^b^	0.08 ± 0.01 ^b^	0.08 ± 0.01 ^b^	0.08 ± 0.01 ^b^	0.08 ± 0.01 ^b^	0.10 ± 0.02 ^a^	0.08 ± 0.01 ^b^	0.09 ± 0.02 ^ab^	0.09 ± 0.02 ^ab^	0.08 ± 0.01 ^b^
*trans*-C18:3	0.02 ± 0.00	0.02 ± 0.00	0.02 ± 0.00	0.02 ± 0.00	0.02 ± 0.00	0.02 ± 0.01	0.01 ± 0.01	0.02 ± 0.01	0.02 ± 0.01	0.02 ± 0.01
Ʃ PUFA	5.85 ± 0.01 ^b^	5.85 ± 0.02 ^b^	5.87 ± 0.03 ^b^	5.83 ± 0.00 ^b^	5.76 ± 0.08 ^b^	59.49 ± 1.57 ^a^	59.37 ± 0.05 ^a^	59.44 ± 1.57 ^a^	59.43 ± 1.48 ^a^	58.62 ± 0.16 ^a^

Different letters in same row indicate significant differences (*p* < 0.05) per Tukey’s test.

## Data Availability

Research data are available in the article.

## Abbreviations

The following abbreviations are used in this manuscript:

ANOVA	Analysis of variance
BHT	Butylated hydroxytoluene
GAE	Gallic acid equivalents
LO	Low oleic oil
HO	High oleic oil
SO	Sunflower oil
HPP	High-pressure processing
SAFA	Saturated fatty acid
MUFA	Monounsaturated fatty acid
PUFA	Polyunsaturated fatty acid

## Appendix A

**Table A1:** Proximal composition of tomato by-product according to harvesting season (S1: 2022; S2: 2023; S3: 2024).

Determination	Method	S1 [43]	S2 [24]	S3
Moisture (%)	ISO 712:2009 [44]	65.15	84.95	76.57
Crude protein (%)	ISO 20483:2013 [45]	7.25	3.33	3.27
Crude fat (%)	AACC, 2010 [46]	6.32	1.65	3.45
Ashes (%)	ISO 2171:2007 [47]	1.15	0.86	1.00
Dietary fiber (%)	AOAC 93.19:2000 [48]	20.13	9.21	15.71
Lycopene (µg/g DW)	Silva et al. [49]	582.0 ± 12.7	1174.3 ± 43.1	776.7 ± 25.5
Total polyphenols (µg GAE/g DW)	Sánchez-Rangel et al. [50]	290.2 ± 4.6	601.0 ± 15.0	473.8 ± 57.6

**Table A2:** Physical–chemical methods used for the sunflower oil’s characterization.

Determination	Analytical Method
Free fatty acids (acid value)	ISO 660:2020 (g oleic acid/100 g oil) [51]
Composition of fatty acids by gas chromatography	Preparation by ISO 12966-2:2017 [52] and analysis by ISO 12966-4:2015 [53].
Tocopherols by HPLC	ISO 9936:2016 (mg/kg) [54]
Color	Color Space CIE L*a*b*. Device: DigiEye (VeriVide Ltd., Leicester, UK) equipped with a Nikon D90 SLR camera (Nikon Co. Ltd., Tokyo, Japan). Illuminant: Artificial Daylight F18 T8/D65 (VeriVide Ltd., Leicester, UK). Associate software: DigiEye v2.80 (VeriVide Ltd., Leicester, UK)
